# Comparison of three cognitive assessment methods in post-stroke aphasia patients

**DOI:** 10.3389/fpsyg.2022.896095

**Published:** 2022-10-20

**Authors:** Zhijie Yan, Shuo Xu, Dongshuai Wei, Xinyuan He, Chong Li, Yongli Zhang, Mengye Chen, Jingna Zhang, Xiaofang Li, Qing Yang, Jie Jia

**Affiliations:** ^1^Department of Rehabilitation Medicine, Huashan Hospital, Fudan University, Shanghai, China; ^2^Department of Rehabilitation Medicine, The Third Affiliated Hospital of Xinxiang Medical University, Xinxiang, China; ^3^National Clinical Research Center for Aging and Medicine, Huashan Hospital, Fudan University, Shanghai, China

**Keywords:** post-stroke aphasia, fluent aphasia, non-fluent aphasia, cognitive impairment, NLCA

## Abstract

**Background:**

The cognitive level of post-stroke aphasia (PSA) patients is generally lower than non-aphasia patients, and cognitive impairment (CI) affects the outcome of stroke. However, for different types of PSA, what kind of cognitive assessment methods to choose is not completely clear. We investigated the Montreal Cognitive Assessment (MoCA), the Mini-Mental State Examination (MMSE), and the Non-language-based Cognitive Assessment (NLCA) to observe the evaluation effect of CI in patients with fluent aphasia (FA) and non-fluent aphasia (NFA).

**Methods:**

92 stroke patients were included in this study. Demographic and clinical data of the stroke group were documented. The language and cognition were evaluated by Western Aphasia Battery (WAB), MoCA, MMSE, and NLCA. PSA were divided into FA and NFA according to the Chinese aphasia fluency characteristic scale. Pearson’s product–moment correlation coefficient test and multiple linear regression analysis were performed to explore the relationship between the sub-items of WAB and cognitive scores. The classification rate of CI was tested by Pearson’s Chi-square test or Fisher’s exact test.

**Results:**

The scores of aphasia quotient (AQ), MoCA, MMSE, and NLCA in NFA were lower than FA. AQ was positively correlated with MoCA, MMSE, and NLCA scores. Stepwise multiple linear regression analysis suggested that naming explained 70.7% of variance of MoCA and 79.9% of variance of MMSE; comprehension explained 46.7% of variance of NLCA. In the same type of PSA, there was no significant difference in the classification rate. The classification rate of CI in NFA by MoCA and MMSE was higher than that in FA. There was no significant difference in the classification rate of CI between FA and NFA by NLCA.

**Conclusion:**

MoCA, MMSE, and NLCA can be applied in FA. NLCA is recommended for NFA.

## Introduction

Stroke is a common clinical cerebrovascular disease. The research data show that there were 80.1 million stroke cases worldwide in 2016 (GBD 2016 Stroke Collaborators, 2019). In China, the incidence of stroke in 2030 is estimated to be 1.5 times that in 2010 (Wang et al., 2020). Post-stroke aphasia (PSA) is an acquired functional defect mainly manifested in the disorder of language output and reception process after the damage of the central nervous system (Stefaniak et al., 2020), accounting for about one-third of the total stroke population (Flowers et al., 2016). The increasing number of patients who experienced stroke events also showed an increasing trend of PSA. The interaction exists in language and cognitive function (Ardila and Rubio-Bruno, 2018). Some studies have shown that the cognitive function after stroke is related to the impairment of language function, and the cognitive level of PSA is generally lower than that of non-aphasia patients (Kang et al., 2016; Yao et al., 2020). As an independent predictor, cognitive function also can judge the prognosis of stroke and affect the rehabilitation outcome of stroke (Kwon et al., 2020).

Cognition is the general name of the process of recognizing and knowing things, including perception, recognition, attention, memory, concept formation, thinking, reasoning, and image process. It belongs to the high-level activities of the cerebral cortex. After brain injury such as stroke, the function of the cerebral cortex is affected to varying degrees, resulting in cognitive impairment (CI; Norris et al., 2016). An objective and comprehensive evaluation of cognitive function is helpful to formulate targeted treatment plans and effectively improve patients’ cognitive function (Cicerone et al., 2019). Patients with PSA often have CI, and more than half of patients with aphasia also have nonverbal CI (Fonseca et al., 2019). Previous studies have confirmed that cognitive function, including nonverbal CI, is associated with language impairment (Bonini and Radanovic, 2015; Ardila and Rubio-Bruno, 2018; Yao et al., 2020). However, there is no clear conclusion about the degree of correlation between CI and language damage and what language factors affect it.

In the previous literature, there have been studies focusing on the evaluation of some dimensions of cognitive function, such as execution, attention, memory, and so on (Murray, 2012; Pompon et al., 2015; Thompson et al., 2018; Simic et al., 2019). The results of some studies comparing the effectiveness of these measures, such as the Montreal Cognitive Assessment (MoCA) and the Mini-Mental State Examination (MMSE), in the evaluation of cognitive function after stroke show that MoCA may be more valid in post-stroke cognitive screening (Dong et al., 2010; Burton and Tyson, 2015). However, because the cognitive assessment tools used in previous studies such as MoCA and MMSE contain a large number of language components, although the evaluation results show that PSA patients have CI, it is still difficult to distinguish between real cognitive problems and language communication problems. To better measure the cognitive level of patients with language dysfunction, respond to a more real and accurate functional state and formulate a more suitable treatment plan for patients, it is necessary to study the application of nonverbal cognition in PSA. In recent years, some scholars have made efforts in the neural basis, clinical characteristics, evaluation, and treatment of nonverbal cognition (Peach, 2017; Wu et al., 2017; Schumacher et al., 2019; Yao et al., 2020).

This study aims to compare the three cognitive assessment methods, including the non-language-based Cognitive Assessment (NLCA), MoCA, and MMSE, and provide suggestions for the evaluation of CI in patients with PSA.

## Materials and methods

### Study population

A total of 92 stroke inpatients from Huashan Hospital, Fudan University, were recruited between May 2021 and June 2022. In this study, subjects were divided into non aphasia (NA) and aphasia, and aphasia patients were subdivided into fluent aphasia (FA) and non-fluent aphasia (NFA). Combined with symptoms, medical history, and imaging data, stroke patients with the aphasia quotient (AQ) of Western Aphasia Battery (WAB) lower than 93.8 points were judged as PSA (Kertesz and Poole, 2004). Among patients with PSA, those who scored 21 to 27 on the Chinese aphasia fluency characteristics scale were judged as FA, and those with a score of 9 to 13 are judged as NFA (Gao, 2006).

The inclusion criteria for PSA were as follows: (a) right handedness, (b) Chinese as the first language, (c) first stroke, (d) left hemisphere lesions, (e) AQ < 93.8, (f) can cooperate to complete all assessments and sign informed consent. The inclusion criteria of NA was that AQ ≥ 98.4, and other conditions were the same as above. On the basis of PSA, according to the score of the Chinese aphasia fluency characteristics scale, 21 to 27 points were the inclusion criteria for FA, and 9 to 13 points were the inclusion criteria for NFA. The exclusion criteria were as follows: (a) recurrent stroke, (b) CI caused by other causes or existing before stroke, such as Alzheimer’s disease, (c) cerebellar and brainstem lesions or severe dysarthria, (d) severe audiovisual impairment, and (e) other serious medical diseases or unstable conditions.

This study was performed in line with the principles of the Declaration of Helsinki. Approval was granted by the Ethics Committee of Huashan Hospital, Fudan University [ethical approval no. (2021) Linshen No. (503)]. Signed written informed consent was given by all participants or their legal representatives.

### Measurement methods

In this study, WAB was used to judge whether it was PSA and the severity of PSA, and the Chinese aphasia fluency characteristics scale was used to judge fluency. The MoCA, MMSE, and NLCA were selected to screen and evaluate the cognitive function.

WAB was published by Andrew et al. in 1974. The severity of language impairment was assessed by AQ calculated from the sub-item scores of spontaneous, comprehension, repetition, and naming. The full score of AQ was 100 points, and < 93.8 points could be used to judge aphasia. The lower the score, the more serious the language impairment (Kertesz and Poole, 2004). The Chinese aphasia fluency characteristics scale evaluated 9 spoken feathers, including vocabulary, intonation, pronunciation, length of phrase, laborsome speech, press of speech, substantive words, grammar, and paraphasia. The possible scores of each item were 1, 2, and 3, and the total score of the scale was 9 to 27. According to the total score, we judged whether aphasia patients were FA (21–27) or NFA (9–13; Gao, 2006).

MoCA was developed by Dr. Nasreddine in 1996 and officially published in English and French in 2005. The scale takes about 10 min and has reliable results. It can sensitively screen mild CI and mild Alzheimer’s disease (Nasreddine et al., 2005). MoCA Beijing edition was used in this study. The evaluation contents include visual space and executive function, naming, memory, attention, language, abstraction, delayed recall, and orientation. The full score is 30 points and ≥ 26 points are normal. MMSE was developed by Folstein et al. in 1975 and Galasko et al. developed a simplified version in 1990. It is mainly used for the evaluation of CI in patients with dementia. It takes about 5–15 min. The evaluation contents include orientation, memory, attention and computing power, and language ability. The full score is 30 and ≥ 27 is normal (Folstein et al., 1975). NLCA was developed by Xiaojia Liu and others in 2013. After using NLCA to evaluate aphasia patients, mild CI patients, and normal people, the scale’s reliability, effectiveness, and practicability are confirmed. The scale mainly relies on visual materials to evaluate five nonverbal cognitive dimensions, including visuospatial function, attention, memory, logical reasoning ability, and executive function, with a full score of 80, ≥ 75 points is normal (Wu et al., 2017).

All the evaluation contents were completed by two uniformly trained speech-language therapists within 3 days after recruitment.

### Statistical analysis

Statistical analyses were conducted using SPSS 25.0 (IBM Corporation, Armonk, NY, United States). For two groups of continuous numerical variables that conform to the normal distribution, the Student’s *t-*test is used, which is expressed by mean ± standard deviation. The mean values of three groups of continuous numerical variables were compared using one-way ANOVA. If it does not conform to the normal distribution, a nonparametric test shall be adopted. Categorical variables were expressed by rate, using Pearson’s Chi-square test or Fisher’s exact test. In the general characteristics, age, years of education, and course of disease were statistically analyzed by one-way ANOVA, and gender was analyzed by Pearson’s Chi-square test. In PSA, the scores of AQ, spontaneous, comprehension, repetition, and naming were analyzed by Mann–Whitney’s U test, and the scores of MOCA, MMSE, and NLCA were analyzed by *t*-test. The classification rate of CI was analyzed by Pearson’s Chi-square test or Fisher’s exact test. Pearson’s product–moment correlation coefficient test was used to explore the correlation between variables. All variables which demonstrated significant moderate or higher correlations (*r* > 0.3, *p* < 0.01) were entered into stepwise multiple linear regression analysis to evaluate their potential impacts on cognitive function evaluation in PSA. A two-sided *p* < 0.05 was considered to be statistically significant in this study.

## Results

### General characteristics

**Figure 1:**
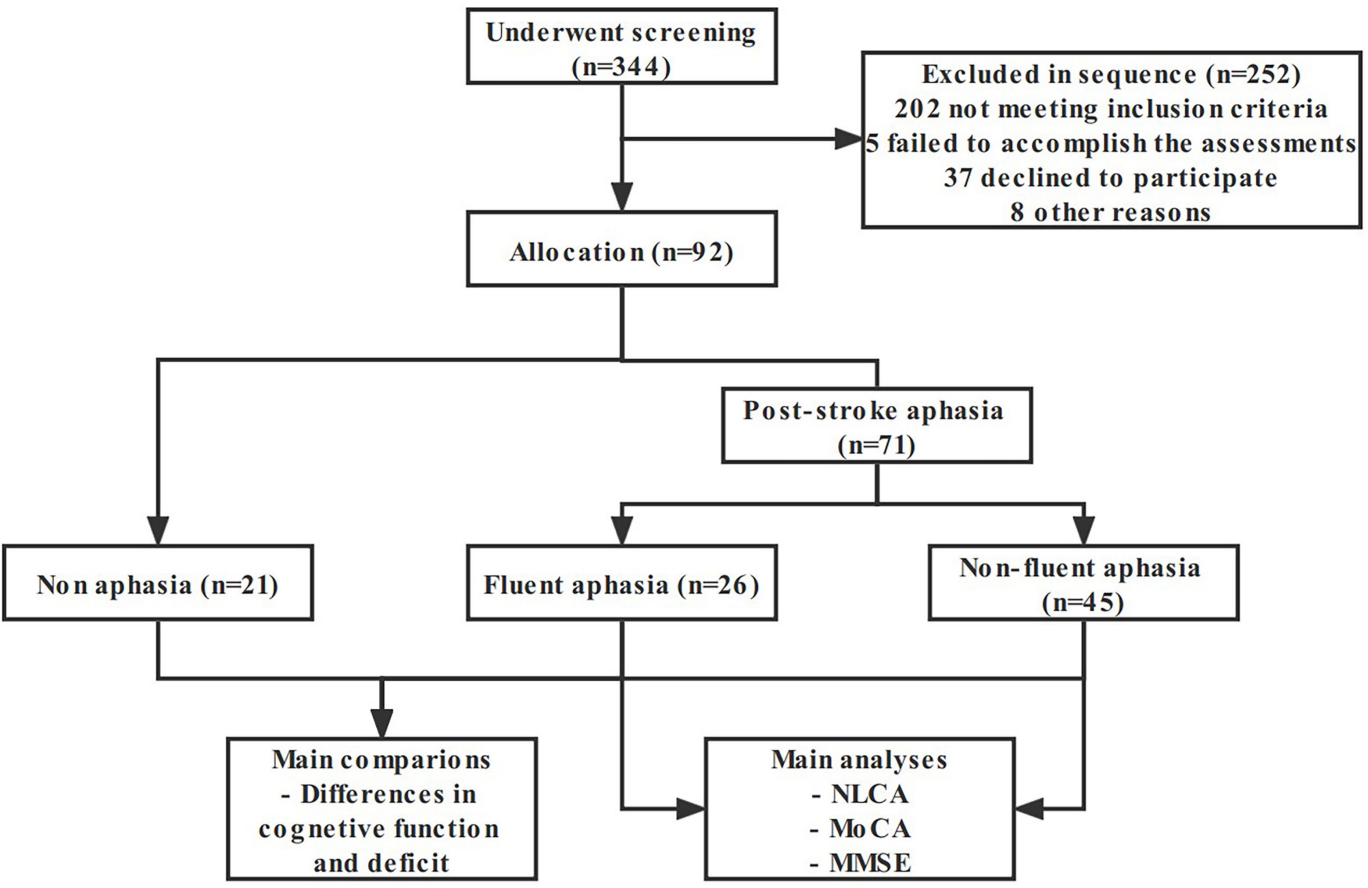
Flow chart of the study sample and procedures. *NLCA* the Non-language-based Cognitive Assessment, *MoCA* the Montreal Cognitive Assessment, *MMSE* the Mini-Mental State Examination.

This study screened 344 patients, of which 252 were excluded and 92 entered the analysis procedure (Figure 1). The characteristics of subjects are presented in Table 1. There was no significant statistical difference in age, gender, education, and course of disease between groups (*p* = 0.487, 0.474, 0.511, and 0.571, respectively).

**Table 1:** Characteristics of all subjects.

	NA (n = 21)	FA (n = 26)	NFA (n = 45)	*p* value
Age (years)	55.86 ± 15.048	57.19 ± 14.924	59.93 ± 12.520	0.487^a^
Gender				0.474^b^
Female	8(38.1)	9(34.6)	22(48.9)	
Male	13(61.9)	17(65.4)	23(51.1)	
Education (years)	13.24 ± 3.145	12.88 ± 3.192	12.29 ± 3.375	0.511^a^
Post-stroke duration (months)	11.48 ± 39.011	4.92 ± 9.629	8.31 ± 12.606	0.571^a^

Data are expressed as mean ± SD or number of observations (% of total observation). NA non aphasia, FA fluent aphasia, NFA non-fluent aphasia.

a 1-way analysis of variance.

b Pearson’s Chi-square test.

### Language and cognitive assessments

All subjects received WAB, MOCA, MMSE, and NLCA tests. WAB is to assess the severity of aphasia, while MOCA, MMSE, and NLCA are to assess CI. The scores of AQ, spontaneous, comprehension, repetition, naming, MOCA, MMSE and NLCA in FA were significantly higher than those in NFA (*p* < 0.001; *p* < 0.001; *p* < 0.001; *p* < 0.001; *p* < 0.001; *p* < 0.001; *p* < 0.001; *p* = 0.001, respectively; Table 2). Compared with FA, NFA seems to have more serious impairment of language function and cognitive function. As shown by the results in Table 3, MoCA and MMSE are positively correlated with all aspects of language assessment, while NLCA and repetition are not. Multiple linear regression analysis showed that naming could explain 70.7% of variance of MOCA and 79.9% of variance of MMSE; comprehension explained 46.7% of variance of NLCA. Language dysfunction has affected the three screening tools of CI to varying degrees.

**Table 2:** The scores of WAB, MOCA, MMSE, and NLCA of FA and NFA.

	FA (n = 26)	NFA (n = 45)	*p* value
AQ	88.1000 ± 8.4639	33.022 ± 25.0909	<0.001^a^
Spontaneous Comprehension Repetition Naming	16.65 ± 2.652 181.81 ± 29.732 96.62 ± 5.216 84.73 ± 14.584	4.67 ± 4.028 114.27 ± 76.920 50.27 ± 77.954 21.56 ± 31.017	<0.001^a^ <0.001^a^ <0.001^a^ <0.001^a^
MOCA total score	17.15 ± 6.915	4.13 ± 5.643	<0.001^b^
MMSE total score	22.15 ± 6.259	7.60 ± 7.472	<0.001^b^
NLCA total score	54.962 ± 21.0081	38.233 ± 27.9097	0.001^b^

Data are expressed as mean ± SD. FA fluent aphasia, NFA non-fluent aphasia, AQ aphasia quotient, MOCA the Montreal Cognitive Assessment, MMSE the Mini-Mental State Examination, NLCA the Non-language-based Cognitive Assessment.

a Mann–Whitney’s U test.

b Student’s *t*-test.

**Table 3:** The correlation among AQ, spontaneous, comprehension, repetition, naming, MOCA, MMSE, and NLCA.

	AQ	Spontaneous	Comprehension	Repetition	Naming
MOCA	0.830^***a^	0.805^***a^	0.673^***a^	0.348^**a^	0.844^***a^
MMSE	0.889^***a^	0.835^***a^	0.710^***a^	0.475^***a^	0.896^***a^
NLCA	0.514^***a^	0.459^***a^	0.689^***a^	0.087^*a^	0.460^***a^

Data are expressed as correlation coefficient. AQ aphasia quotient, MOCA the Montreal Cognitive Assessment, MMSE the Mini-Mental State Examination, NLCA the Non-language-based Cognitive Assessment.

* *p* > 0.05;

** *p* < 0.01;

*** *p* < 0.001.

a Pearson’s product–moment correlation coefficient test.

### The classification rate of CI

For NA, there was no statistical difference in the classification rate of CI obtained by using the three cognitive measurement methods. In the same type of PSA, there was no significant difference in the classification rate of CI between the three methods (*p* = 0.153, 0.546; Table 4). The classification rate of CI in NFA using MOCA and MMSE was higher than that in FA (*p* = 0.015, *p* = 0.001). Nevertheless, there was no significant difference in the classification rate of CI between FA and NFA using NLCA (*p* = 0.182; Table 5).

**Table 4:** The classification rate of CI of MOCA, MMSE, and NLCA in NA, FA, and NFA.

	NA			FA			NFA		
	NCI	CI	*p* value	NCI	CI	*p* value	NCI	CI	*p* value
MOCA	10(47.6)	11(52.4)		4 (15.4)	22 (84.6)		0(0)	45(100)	
MMSE	15(71.4)	6(28.6)		9 (34.6)	17 (65.4)		2(4.4)	43(95.6)	
NLCA	10(47.6)	11(52.4)		4 (15.4)	22 (84.6)		2(4.4)	43(95.6)	
			0.241 ^a^			0.153 ^a^			0.546 ^b^

Data are expressed as the number of observations (% of total observation). NA non aphasia, FA fluent aphasia, NFA non-fluent aphasia, MOCA the Montreal Cognitive Assessment, MMSE the Mini-Mental State Examination, NLCA the Non-language-based Cognitive Assessment, NCI non-cognitive impairment, CI cognitive impairment.

a Pearson’s Chi-square test.

b Fisher’s exact test.

**Table 5:** The classification rate of CI in FA and NFA by MOCA, MMSE and NLCA.

	MOCA			MMSE			NLCA		
	NCI	CI	*p* value	NCI	CI	*p* value	NCI	CI	*p* value
FA	4(15.4)	22(84.6)		9 (34.6)	17 (65.4)		4 (15.4)	22 (84.6)	
NFA	0(0)	45(100)		2 (4.4)	43 (95.6)		2 (4.4)	43 (95.6)	
			0.015 ^a^			0.001 ^a^			0.182 ^a^

Data are expressed as the number of observations (% of total observation). FA fluent aphasia, NFA non-fluent aphasia, MOCA the Montreal Cognitive Assessment, MMSE the Mini-Mental State Examination, NLCA the Non-language-based Cognitive Assessment, NCI non-cognitive impairment, CI cognitive impairment.

a Fisher’s exact test.

## Discussion

In this study, three representative cognitive assessment methods were selected to study whether language impairment in stroke patients has an impact on cognitive screening, and provide reference suggestions for cognitive assessment of post-stroke aphasia patients. As for the relationship between language and cognition, there may be overlapping areas at the neural level, such as the frontal lobe, temporal lobe, and parietal lobe, and the damage of language leads to the common damage of cognitive-related brain networks (Schumacher et al., 2019). The behavioral results of this study showed that the total scores of the three cognitive assessment methods were positively correlated with AQ, which showed that language impairment had an impact on different cognitive assessment methods. The previous studies have also shown a link between language impairment and cognitive impairment (Bonini and Radanovic, 2015; Ardila and Rubio-Bruno, 2018; Fonseca et al., 2019; Yao et al., 2020). The results showed that the language and cognitive impairment of NFA was more serious in stroke population. Therefore, the choice of cognitive assessment for such patients should be more cautious. We preliminarily analyzed the components of the scales. Except for visual space and executive function in MoCA, other test items rely on language expression ability. In the total score of 30 points, MOCA has 24 points and MMSE has 25 points, which needs language expression. The NLCA evaluation process can be completed with the help of audio-visual perception, without oral expression. However, the previous behavioral research results also support that nonverbal CI is related to comprehension impairment (Caplan et al., 2013; Thompson et al., 2018; LaCroix et al., 2021). This shows that nonverbal cognitive assessment methods reduce the impact of language impairment on the evaluation of cognitive function, although they may still be unable to completely get rid of the interaction between language and cognition.

For non-aphasia patients after stroke, MoCA can be recommended for cognitive screening, which is consistent with the previous research results (Dong et al., 2010; Burton and Tyson, 2015). In PSA, there was no significant difference in the classification rate of CI among MOCA, MMSE, and NLCA, but compared with NLCA, the classification rate of CI in NFA using MOCA and MMSE was higher than that in FA. Compared with NLCA, MOCA and MMSE may overestimate the degree of CI in patients with NFA due to language deficit. The results of analysis showed that NLCA is less affected by language factors. Scholars developed NLCA and other nonverbal cognitive evaluation scales for more truly reflecting the cognitive level of aphasia patients. However, NLCA and other nonverbal cognitive evaluation scales have the disadvantages of being time-consuming compared with MOCA, MMSE, and other scales. Is it necessary to use nonverbal cognitive assessment for all PSA? To balance various factors, given the results of this study, we recommend the use of nonverbal cognitive evaluation tools for NFA, and MoCA, MMSE, and NLCA can be applied in FA. In addition, we also analyzed the Barthel Index. The post-test results of ANOVA showed that there was a statistical difference between NA and NFA. This may indicate that NFA also has great obstacles in daily life activities, not only in language and cognition, but also may be a direction of future research.

The current research has some limitations. First, the classification of PSA is not comprehensive enough. In this study, PSA was only classified into two categories, not eight categories. In the future, we can expand the sample size based on previous studies for a more detailed classification of aphasia. Second, the evaluation result of NLCA has only a critical value without severity classification. Therefore, the classification of the severity of CI needs the help of other tools. Third, this study focuses on the results of the behavioral evaluation and does not involve imaging, such as MRI. In the future, imaging can be used to explore the neural mechanism between language and cognitive impairments, to provide the basis for the integrated rehabilitation of language and cognition.

## Conclusion

The impairments of language and cognitive function in patients with NFA are more serious than those in patients with FA. The results of the cognitive assessment were positively correlated with language impairment. MoCA, MMSE, and NLCA can be applied to FA, and NLCA is more recommended to be used in NFA.

## Data availability statement

The raw data supporting the conclusions of this article will be made available by the authors, without undue reservation.

## Ethics statement

The studies involving human participants were reviewed and approved by the Ethics Committee of Huashan Hospital, Fudan University [ethical approval no. (2021) Linshen No. (503)]. The patients/participants provided their written informed consent to participate in this study.

## Author contributions

ZY, XL, and JJ: conceived and designed the analysis. ZY, SX, and JZ: collected the data. ZY, SX, DW, XH, CL, YZ, MC, and QY: contributed data or analysis tools. ZY and XL: performed the analysis. ZY and JJ: writing—original draft: All authors discussed the results, contributed to the final manuscript and writing—review and editing. ZY and SX contributed equally to this work and are the first authors. JJ is the corresponding author of this article. QY is the co-corresponding author. All authors contributed to the article and approved the submitted version.

## Funding

This work was supported by the National Key R&D Program of China (2018YFC2002300), the National Natural innovation research group project of China (82021002), and the National Natural major research program integration project of China (91948302).

## Conflict of interest

The authors declare that the research was conducted in the absence of any commercial or financial relationships that could be construed as a potential conflict of interest.

## Publisher’s note

All claims expressed in this article are solely those of the authors and do not necessarily represent those of their affiliated organizations, or those of the publisher, the editors and the reviewers. Any product that may be evaluated in this article, or claim that may be made by its manufacturer, is not guaranteed or endorsed by the publisher.
